# Biodegradable TPS/PBAT Blown Films with Ascorbyl Palmitate and Sodium Ascorbyl Phosphate as Antioxidant Packaging

**DOI:** 10.3390/polym16233237

**Published:** 2024-11-22

**Authors:** Rosi Andini Arumsari, Phanwipa Wongphan, Nathdanai Harnkarnsujarit

**Affiliations:** 1 Department of Packaging and Materials Technology, Faculty of Agro-Industry, Kasetsart University, 50 Ngam Wong Wan Rd., Latyao, Chatuchak, Bangkok 10900, Thailand; rosiandini.a@ku.th (R.A.A.); phanwipa.w@ku.th (P.W.); 2 Center for Advanced Studies for Agriculture and Food, Kasetsart University, 50 Ngam Wong Wan Rd., Latyao, Chatuchak, Bangkok 10900, Thailand

**Keywords:** active packaging, ascorbyl palmitate, antioxidant, polybutylene adipate-co-terephthalate, sodium ascorbyl phosphate, thermoplastic starch

## Abstract

The development of biodegradable active packaging is a relevant topic demanding the development of film properties, biodegradability, and the potential to preserve food quality. This study aimed to develop thermoplastic starch (TPS) blended with polybutylene adipate-co-terephthalate (PBAT) films via blown-film extrusion containing ascorbyl palmitate (AP) and sodium ascorbyl phosphate (SAP) as antioxidants. The morphology, mechanism, and barrier and antioxidant properties of the films were analyzed to determine the presence of AP, SAP, and their interaction effect on the film properties. SEM showed that increasing AP and SAP content increased fibrous-like morphology, improving the TPS dispersion. AP slightly decreased mechanical properties, while SAP increased the tensile properties and seal strength of the films. All of the YM values were increased by adding AP and SAP content. The addition of AP and SAP content enhanced the interaction with TPS/PBAT networks due to increasing C-O stretching of ester bonds, compatibility, and hydrophobicity of the polymer. Both water vapor and the oxygen barrier were insignificantly affected by AP and SAP up to 1%, while the permeabilities greatly increased at higher AP and SAP contents due to non-homogeneous and void spaces between the film matrix. TPS/PBAT containing AP and SAP (≥0.5%) effectively enhanced antioxidant capacity in 95% ethanol as a food simulant and reduced the UV light transmission of the films. Finding, the interaction between AP, SAP, and TPS/PBAT matrices effectively changed the microstructures and properties as functionalized antioxidant biodegradable packaging.

## 1. Introduction

Sustainability trends are driving the adoption of eco-friendly packaging as an alternative to conventional packaging made from fossil-based and non-biodegradable materials. These biodegradable plastic materials also promote sustainability and quality in the food industry. The bio-circular green (BCG) economic model system has the potential to enhance competitiveness and respond to the growing consumer demand for eco-friendly packaging by increasing the value added of the resources [1,2,3]. Starch is a highly abundant biodegradable natural polymer. Its potential to be transformed from starch powder into thermoplastics, known as thermoplastic starch (TPS) has led to its widespread use in commercial-scale packaging. This is due to its low production cost and reduced environmental footprint. However, TPS suffers from limitations such as low mechanical strength, high stiffness, low formability, and low stability. It also has a highly hydrophilic nature, stemming from its -OH groups [2,3]. Blending starch with other biopolymers that have hydrophobic properties presents a promising approach to improve these properties. Poly(butylene adipate-co-terephthalate) (PBAT) is a widely used biodegradable polymer in the industry due to its high flexibility, favorable processability, and high hydrophobic properties [3,4,5,6,7,8].

A current development in producing biodegradable packaging involves using compatibilizers, such as antioxidants, to enhance the mechanical and barrier properties as well as the functionality of biodegradable packaging as an active packaging material. The main antioxidants that are usually used in polymer blends and are safe for food are polyphenols, flavonoids (e.g., quercetin), and vitamins [6,9,10,11]. Recent studies on antioxidant packaging have reported that TPS/PBAT film incorporated with quercetin, organically modified montmorillonite, and curcumin effectively slowed the decay of bananas and blueberries and prevented oil degradation. This is due to the developed films possessing high antioxidant properties and suitable molecular permeability [2,11,12,13,14].

Vitamin C, also known as ascorbic acid, is a primary antioxidant commonly used in antioxidant packaging. It offers numerous health benefits, including potent antioxidant, anticancer, and anti-inflammatory properties. Ascorbic acid has been incorporated into polylactic acid (PLA) to create antioxidant packaging [15]. However, its susceptibility to thermal and oxidative degradation, coupled with its lipo-solubility, makes it challenging to maintain its physiological value over extended periods. To address this, structural modifications of ascorbic acid have led to the development of diverse derivatives with enhanced thermal and oxidative stability [9,16,17,18]. Ascorbyl palmitate (AP), a more stable derivative of vitamin C, has been successfully encapsulated in electrospun zein fibers for use as an active coating for butter [19]. Another derivative of vitamin C is sodium ascorbyl phosphate (SAP), which holds significant potential as an antioxidant agent. However, the study of SAP as additives in the packaging is limited. These derivatives of vitamin C have different polarities that possibly enhance the properties of functional active packaging.

The study of incorporating vitamin derivatives into TPS/PBAT blend films remains limited. Given the differing polarity properties of AP and SAP, this research hypothesizes that incorporating these additives into TPS/PBAT blends could modify the morphology and properties of the resulting films, enhancing their potential as antioxidant packaging. This study aims to develop biodegradable active packaging by compounding AP and SAP with TPS/PBAT via blown-film extrusion. The findings are expected to contribute to the development of advanced biodegradable active packaging.

## 2. Materials and Methods

### 2.1. Materials

Native cassava starch powders were purchased from Siam Modified Starch Co., Ltd., Pathumthani, Thailand. Commercial grade glycerol (C_3_H_8_O_3_, SAC SCI-ENG Ltd., Bangkok, Thailand) was used as the plasticizer. PBAT (Ecoflex^®^F Blend C1200) was purchased from BASF, Ludwigshafen, Germany. Ascorbyl palmitate powders were purchased from Value Industrial Products Co., Ltd., Bangkok, Thailand and sodium ascorbyl phosphate powders were purchased from CHEMIPAN Corp., Bangkok, Thailand. Trolox, 2,2-diphenyl-1-picrylhydrazyl (DPPH^•^) and ethanol 99% were all analytical grade and purchased from Sigma-Aldrich Corp., Saint Louis, MO, USA.

### 2.2. Film Preparation

Native starch was dried in a hot air oven at 50 °C overnight to remove moisture. Starch powders were mixed with glycerol at a starch-to-glycerol ratio of 100:35 using a dough mixer (SC-236A, Stelang Electric Appliance Co., Ltd., Foshan, China) for 10 min. Antioxidant compounds, including ascorbyl palmitate (AP) and sodium ascorbyl phosphate (SAP), were incorporated at varying concentrations of 0.5%, 1%, and 3% AP, 0.5% and 1% SAP, and mixtures of 0.5%/0.5% and 2%/1% AP/SAP. These mixtures were compounded into thermoplastic starch (TPS) using a twin-screw extruder (Labtech Engineering, Samut Prakarn, Thailand) with an L/D ratio of 40 and a screw diameter of 20 mm. The temperature profile was set at 85–165 °C with a screw speed of 280 rpm. The resulting AP-, SAP-, and AP-SAP-compounded TPS pellets were cut into 2.5 mm pieces. PBAT and TPS containing AP and SAP pellets were compounded at weight ratios of PBAT:TPS of 50:50. The pelleted compounds were mixed using a twin-screw extruder with a temperature profile of 85–155 °C at a screw speed of 200 rpm. The blended materials were cut into 2.5 mm pellets and kept in Ziploc bags.

Blended pellets were converted into films by a blown-film single-screw extruder with an L/D ratio of 30 and a screw diameter of 25 mm (Labtech Engineering, Samut Prakan, Thailand) with a temperature profile of 145–165 °C. The screw speed and nip roll speed were 29–35 and 2.2–2.4 rpm, respectively. Films were kept in Ziploc bags until testing. Thickness was determined using a 547–401 Absolute Thickness Gauge (Mitutoyo, Kanagawa, Japan).

### 2.3. Scanning Electron Microscopy (SEM)

The microstructure of the samples was determined using a scanning electron microscope (SEM) (FEI Quanta 450, Thermo Fisher Scientific, Waltham, MA, USA) at an accelerating voltage of 15 kV. Samples were immersed in liquid nitrogen and freeze-fractured before being mounted on a metal stub. The samples were coated with gold using a sputter coater (SPI-Module, Structure Probe, Inc., West Chester, PA, USA) and examined for surface and cross-sectional structures at magnifications of 250× and 1500×, respectively.

### 2.4. Fourier Transform Infrared Spectroscopy (FTIR)

The IR spectra of the film samples were determined using a Bruker Tensor 27 FT-IR Spectrometer (Bruker OPTIK GmbH, Ettlingen, Germany). IR absorbance spectra were recorded from 500 to 4000 cm^−1^ at a resolution of 4 cm^−1^ using attenuated total reflection (ATR) mode. An air spectrum was used for background correction.

### 2.5. Mechanical Properties

Tensile strength (TS), elongation at break (EB), and Young’s modulus were measured according to ASTM D882-12 [20] using an Instron universal testing machine (Model 5965, Instron, Norwood, MA, USA). Film samples were cut into 2.5 cm × 10 cm pieces and their thickness was measured five times using a digital micrometer. The films were conditioned in a humidity chamber at 50% relative humidity (RH) and 25 °C for 48 h prior to testing. The films were mounted between the grips at a 5 cm gauge length and tested at a crosshead speed of 500 mm/min. TS and EB values were reported as the average of ten replicates in both the machine direction (MD) and cross-direction (CD).

### 2.6. Heat Sealability

Heat sealability was evaluated using a PARAM™ HST-H3 heat seal tester (Labthink Instruments, Jinan, China) with a 10 mm width seal bar, following the method described by Abdorreza et al. [21] and Das and Chowdhury [22] with slight modifications. Two films were overlapped and heat sealed over 100 mm length under 300 kPa at 115 °C for 1.5 s. Following ASTM F88 [23], the sealed films were cut into 75 mm × 25 mm rectangular strips and conditioned for 48 h at 25 ± 2 °C and 55% RH before testing. The sealing strength was measured using an Instron Universal Testing Instrument model 5965 (Instron, USA) with a 5 kN load cell. Ten heat-sealed specimens were tested.

### 2.7. Barrier Properties

#### 2.7.1. Surface Hydrophobicity

The surface hydrophobicity of the 1 cm × 10 cm films was determined by the contact angle (CA) using a contact angle measurement device (Dataphysics OCA15EC, Dataphysics Instruments GmbH, Filderstadt, Germany). Distilled water (3 μL) was dropped on the film surface using a micro-syringe. Pictures were captured immediately by the SCA 20 program (Dataphysics Instruments GmbH, Filderstadt, Germany) with ten replications.

#### 2.7.2. Water Vapor Permeability (WVP)

The water vapor transmission rate (WVTR) for circular 7 cm diameter films was determined as the average of five replicates using the standard cup method (ASTM E96) [24]. Samples were placed on aluminum cups containing silica gel, covered with an O-ring, and sealed with molten paraffin. Samples were stored in a humidity chamber at 25 °C and 50% RH and weighed daily until a constant weight was reached. The WVTR was calculated from the linear slope between weight and time. The water vapor permeability (WVP) values were calculated using Equation (1).

WVP = (WVTR × L)/ΔP(1)

where L is the film thickness (mm) and ΔP is the difference in water vapor partial pressure (atm) between the two sides of the film.

#### 2.7.3. Oxygen Permeability (OP)

The oxygen transmission rate (OTR) was determined in triplicate following ASTM D3985-051 [25] (ASTM, 2010) using an 8500 Model oxygen permeability analyzer (Illinois Instruments, Inc., Johnsburg, IL, USA) at 23 ± 2 °C and 50% RH. The oxygen permeability (OP) values were calculated using Equation (2).

OP = (OTR × L)/ΔP(2)

where L is the film thickness (mm) and ΔP is the oxygen partial pressure difference (atm) between the two sides of the film.

### 2.8. Light Transmission

Light transmission of 3 cm × 4 cm films was measured three times using a UV-Vis spectrophotometer (Evolution 300, Thermo-Scientific, Waltham, MA, USA). The films were sandwiched between metal plates with a slit, and the percentage of light transmission was recorded at wavelengths ranging from 200 to 800 nm with a scanning speed of 240 nm/min.

### 2.9. Antioxidant Activity

The antioxidant activity of the produced films was determined using the DPPH assay, following a procedure adapted from Panrong et al. [26] with slight modifications. Briefly, film samples were immersed in 20 mL of ethanol for 15 and 30 min at room temperature to measure the release behavior of antioxidant activity. The DPPH assay was based on the method described by Du et al. [27] with minor modifications. A 25 ppm DPPH stock solution was prepared in 100 mL of 99% ethanol. Then, 25 µL of film extract was mixed with 2000 µL of the 25 ppm DPPH ethanol solution in a test tube. After 30 min in the dark, the absorbance was determined at 517 nm using a spectrophotometer (Evolution 300, Thermo-Scientific). The antioxidant capacity was calculated using a Trolox standard curve and the results were expressed as mg TE/0.5g_film sample._ All samples were analyzed in triplicate.

### 2.10. Statistical Analysis

The data were analyzed using SPSS Statistics 27.0 for Windows. An analysis of variance (ANOVA) was used to assess the differences between factors and levels. Tukey’s multiple range test was employed to identify significant differences between group means (*p* < 0.05).

## 3. Results and Discussion

### 3.1. Microstructure

**Figure 1:**
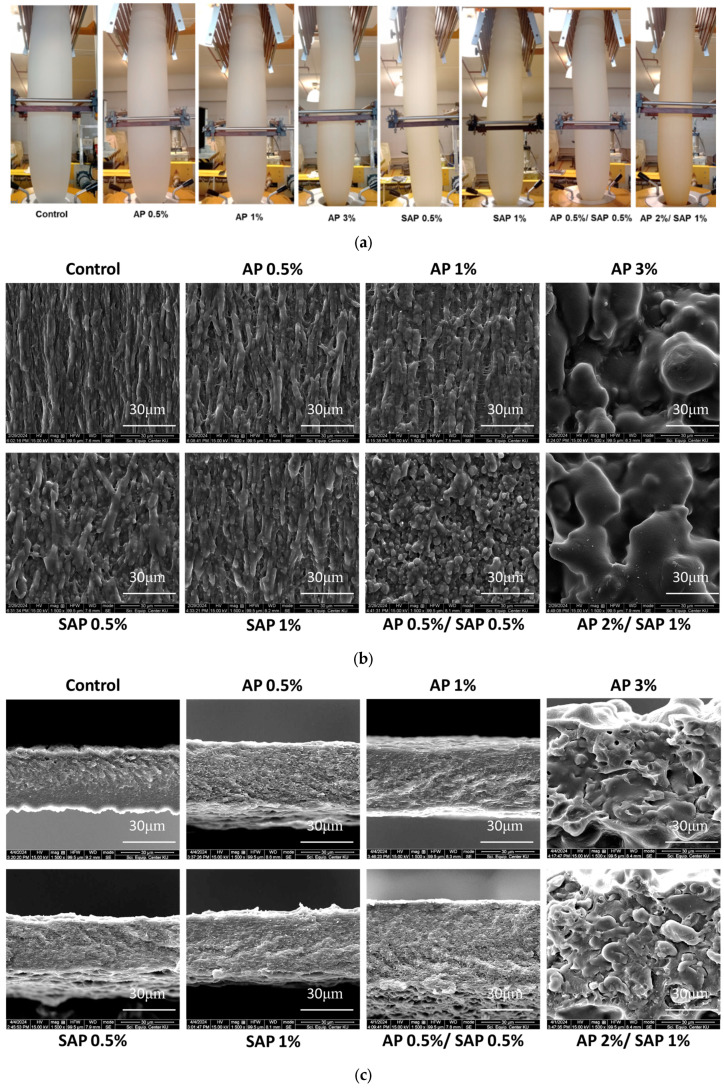
Structures of TPS/PBAT blend films, namely (**a**) appearance during blown-film extrusion; (**b**) surface and (**c**) cross-section containing ascorbyl palmitate (AP) and sodium ascorbyl phosphate (SAP).

TPS/PBAT blend films containing AP and SAP formed continuous tubular structures during the blown extrusion process (Figure 1a), adding both AP and SAP at 0.5% and above slightly increased turbidity of the blown films. The microstructures in the surface and cross-section of the films with varying AP and SAP contents are shown in Figure 1b,c. The TPS/PBAT blend film (control) revealed a fibrous-like surface (Figure 1b). Immiscibility between PBAT and TPS caused phase separation between these polymer blends, giving a dispersed fibrous matrix in the continuous polymer phase. Incomplete melting was observed of the plasticized starch granules commonly formed in the dispersed continuous phase in the PBAT continuous matrix [28]. The incorporation of either AP or SAP at 0.5 and 1% increased the thickness of these fibrous-like matrices. AP and SAP are ascorbic acid derivatives which plasticized the starch and enhanced melting in extrusion [29]. Consequently, the expansion of starch chains due to a higher degree of melting formed larger (thicker) polymer dispersed fibrous chains. Moreover, the edge of the dispersed fibers became clearer with added AP and SAP, suggesting more distinct phase separation. However, the AP 0.5%/SAP 0.5% mixture exhibited a shorter fibrous structure and more dispersed clumps. Results suggested that blending AP and SAP disrupted the networks of the dispersed starch chains. Adding AP, particularly at 1%, slightly enhanced the adhesion between the fibrous phase and the matrix. The amphiphilic nature of AP, with its hydrophilic head and hydrophobic fatty acid tail (palmitate), suggests that it can act as a compatibilizer, enhancing adhesion between starch molecules and the PBAT phase [12,30]. In contrast, SAP0.5% resulted in a thicker fibrous structure than AP0.5%. SAP, being highly soluble in the water–glycerol phase, decomposes into ascorbic acid and sodium phosphate during heat processing, leading to greater starch granule disintegration and network connectivity [31]. High levels of AP and SAP, such as 2% AP/1% SAP and 3% AP, led to extensive melting of the TPS phase, resulting in a continuous interconnected network without any discernible fibrous structures.

Figure 1c shows that the control film exhibited a non-homogeneous matrix with both smoother and rougher regions. The smoother regions likely correspond to PBAT-rich phases, while the rougher regions are attributed to the dispersion of starch polymers within the PBAT matrix. The addition of AP, SAP, and the AP/SAP mixture promoted a more homogeneous dispersion of starch within the polymer matrix. Olivato et al. (2011) [32] reported that the addition of organic acid compatibilizers to TPS/PBAT blends can lead to easier degradation due to chain scission. In contrast, Wongphan et al. (2023) [1] demonstrated that sodium nitrite and sodium erythorbate can act as compatibilizers by disrupting inter- and intra-molecular hydrogen bonds in starch, resulting in denser and more uniform TPS/PBAT blend films. Increasing AP to 1% resulted in the smoothest and most homogeneous structure due to reduced polymer aggregation and improved compatibility between the polymer matrices. In contrast, TPS/PBAT blend films containing SAP exhibited a rougher cross-section with numerous fine pores. This may be attributed to the highly hydrophilic nature of SAP, which can absorb significant amounts of water during processing, leading to shrinkage during blown-film extrusion [33]. Furthermore, higher levels of AP (3%) and AP/SAP mixtures resulted in agglomeration and phase separation in both surface and cross-sectional structures, indicating incompatibility at these concentrations. This behavior is similar to that observed with tartaric acid (TA) in TPS/PBAT blends, where excessive TA levels lead to an increased hydrogen ion (H+) concentration, significantly reducing the molecular weight of TPS and PBAT [34].

### 3.2. FT-IR

**Figure 2:**
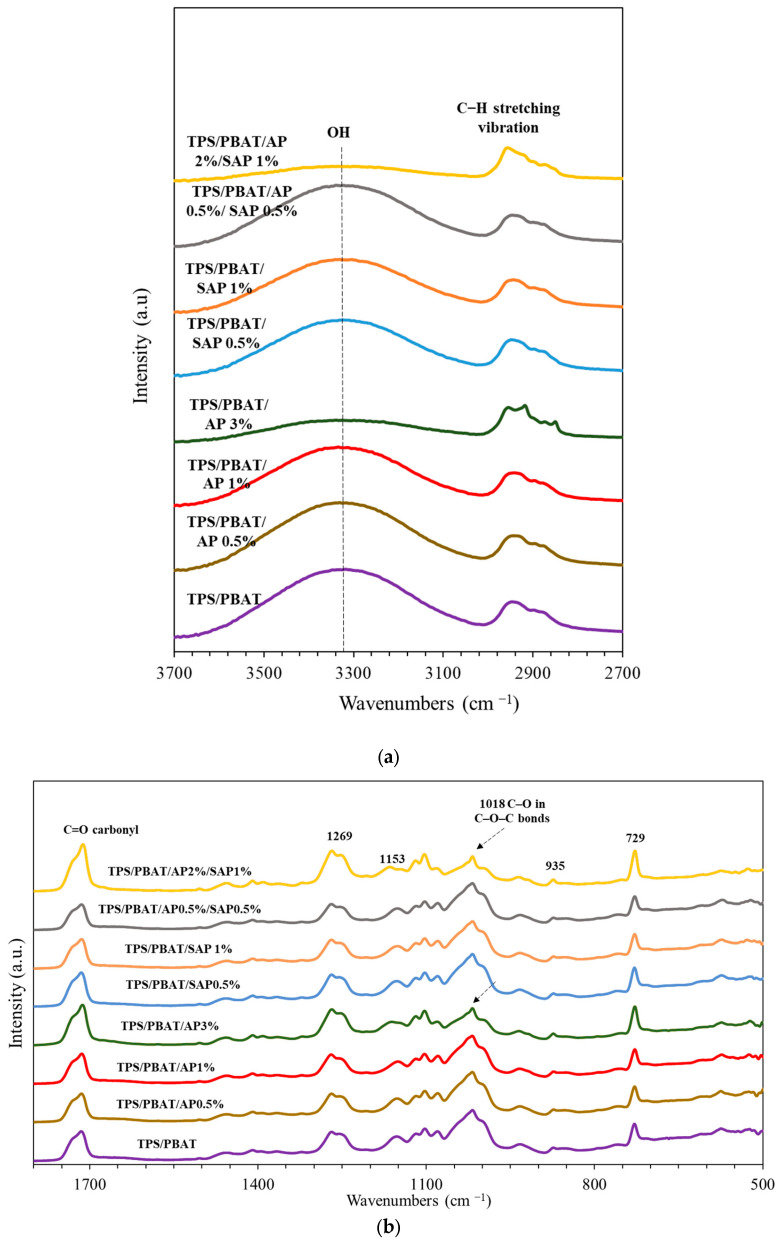
FTIR spectra of TPS/PBAT blend films; (**a**) 2700–3700 cm^−1^; (**b**) 500–1800 cm^−1^, containing ascorbyl palmitate (AP) and sodium ascorbyl phosphate (SAP).

FT-IR analysis revealed major absorption bands between 3300 and 3500 cm^−1^, attributed to inter- and intra-molecular hydrogen bonding in TPS. Upon the incorporation of AP and SAP, these bands shifted to higher wavenumbers (3294–3340 cm^−1^) (Figure 2a), indicating interactions with TPS. However, the intensity of O-H stretching bands significantly decreased in films containing the highest AP concentration (3%) and the AP2%/SAP1% mixture, accompanied by a sharp increase in the intensity of C=O bands around 1715 cm^−1^. This suggests interactions between starch and ascorbic acid derivatives involving C=O groups, leading to the elimination of O-H bonds and a reduction in O-H stretching vibrations [17].

The absorption peak at 2800–3000 cm^−1^ is attributed to the asymmetric and symmetric stretching vibrations of methylene (-CH_2_) and methyl (-CH_3_) groups (Figure 2a). The shape and intensity of theses peaks in this region were influenced by the concentration of AP and SAP. Increasing the amount of AP and SAP in TPS/PBAT films slightly shifted the bands to lower wavenumbers, from 2947 to 2942 and 2944 cm^−1^, respectively. The sensitivity of these bands suggests alterations in the cyclic structure of starch due to atom charge, bond lengths, and force constants. AP and SAP have carbonyl groups that act as proton receptors for hydrogen bonding, possess free hydroxyl groups, and they influence the C–H stretching band between TPS/PBAT blend films [19,33].

The absorption band between 500 and 1500 cm^−1^ represented the fingerprint region of the polymers (Figure 2b). PBAT components presented out-of-plane bending of =C–H in the phenyl ring adsorption at 729 and 873 cm^−1^, C-O symmetric stretching vibrations at 1103 and 1119 cm^−1^, and C-O ester bond stretching at 1269 cm^–1^. Increasing AP up to a 3% concentration increased the intensity of 729 cm^–1^ and slightly shifted to a lower wavenumber, suggesting high interaction between the out-of-plane bending of =C–H in the phenyl ring of PBAT and palmitate acid linkages of AP [33,35,36,37]. Both AP and SAP intensified the peaks at 1018 cm-1, attributed to C-O stretching vibrations of the C-O-C group in the glycosidic linkages of the TPS chain [1,38]. Additionally, peaks at 1153 and 935 cm^−1^, assigned to C-O-C ether stretching, slightly shifted to a lower wavenumber with the incorporation of AP and SAP, suggesting interaction between polymers and additives via the alkyl group of AP and ester linkages of SAP. Additionally, the deformation of phosphate and complexity of phosphate with sodium in SAP possibly showed in the regions of 840–1120 cm^−1^ and 790–900 cm^−1^, respectively [37]. However, the peaks likely merged with the vibrations of the polymers (TPS and PBAT) and were not discernible in Figure 1b. Overall, the results indicate interactions involving C-O stretching vibrations between the ether groups of starch and the ester bonds of the additives.

The peak of 1715 cm^−1^ showed a C=O stretching vibration of the carbonyl group in PBAT. AP 3% and AP2%/SAP 1% increased the intensity of this peak and shifted to lower wavenumbers, at 1713 and 1712 cm^−1^, respectively. This possibly indicates an esterification reaction among the ester moiety of AP and PBAT [1,13,18]. Conversely, SAP had no significant effects on this peak. These findings suggested that AP primarily interacts with the PBAT phase, while SAP primarily interacts with the TPS phase.

### 3.3. Mechanical Properties

**Figure 3:**
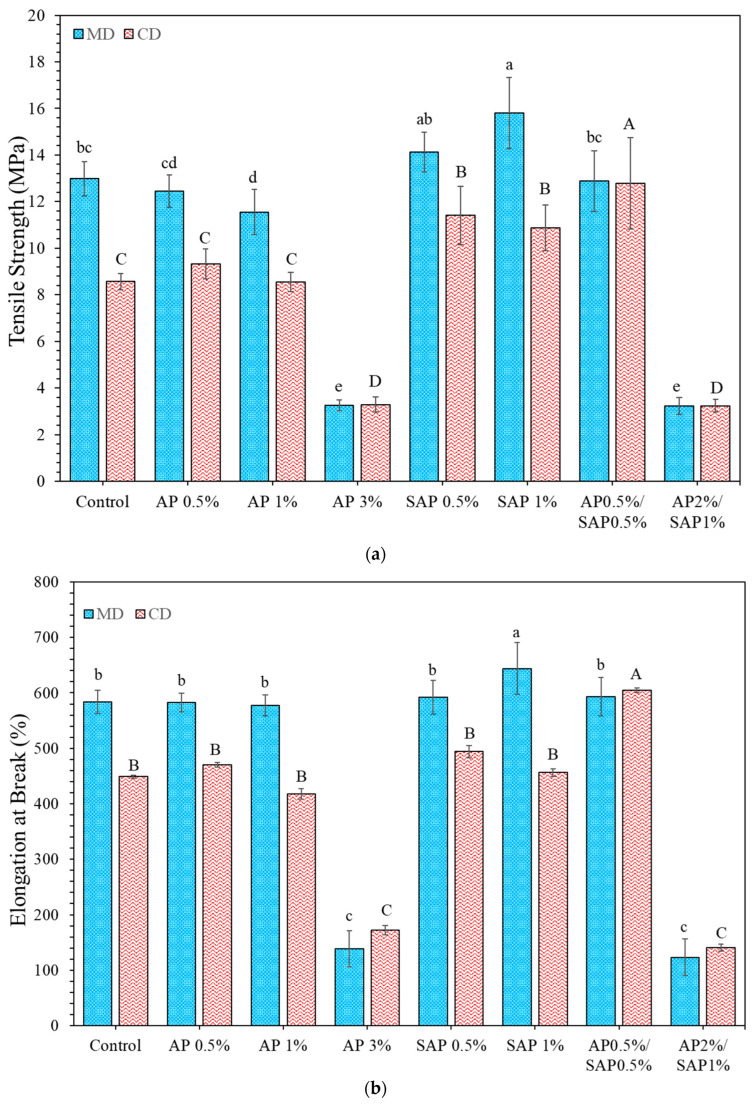

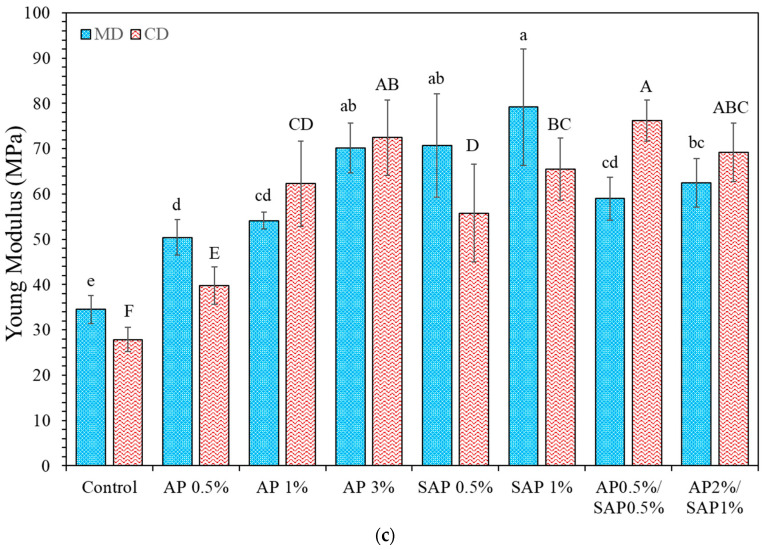
Mechanical properties of (**a**) tensile strength (TS); (**b**) elongation at break (EB); and (**c**) Young’s modulus (YM) on machine direction (MD) and cross-direction (CD) of TPS/PBAT blend film containing ascorbyl palmitate and sodium ascorbyl phosphate. Different upper and lowercase letters indicate significant difference (*p* ≤ 0.05) between samples on CD and MD, respectively.

The mechanical properties of the sample films, including TS, EB, and YM in MD and CD, are presented in Figure 3a–c. The addition of AP up to 1% and the mixture of AP0.5%/SAP 0.5% reduced TS values (Figure 3a). The hydrophilic ascorbic acid and palmitic tail of AP resulted in a homogeneous matrix between TPS and the PBAT phase due to the disruption of the structure, suggesting reduced interfacial adhesion between polymer chains and decreased strength [39]. SAP is highly hydrophilic, easily interacting with the TPS phase and possibly weakening the interaction with the PBAT phase, suggesting increased molecular adhesion within the PBAT matrix. Thus, the TS of TPS/PBAT blend films containing SAP increased as the interfacial adhesion increased.

The EB value of TPS/PBAT blend films incorporated with AP up to 1% slightly decreased by 7% and in MD and CD, respectively (Figure 3b). AP has a hydrophilic and hydrophobic nature, which easily interacts with the TPS/PBAT matrix and reduces the adhesion force. However, low compatibility and low adhesion between the TPS/PBAT films resulted in a lower EB. In contrast, SAP 1% significantly increased the EB value due to compatibility with the TPS phase, which plasticized the TPS and increased stretchability [14].

The highest concentration of AP (3%) and the AP 2%/SAP 1% mixture significantly decreased TS and EB due to the film’s non-homogeneous structure. This was supported by the SEM images, which showed phase separation between PBAT and TPS and agglomeration, indicating poor polymer interconnectivity. Zhang et al. [40] demonstrated that organic acids can generate higher H+ concentrations, leading to a significant decrease in the molecular weight of starch and PBAT. This disruption of the polymer chains can hinder the formation of a good interface between TPS and PBAT, resulting in weaker polymer linkages and, consequently, lower tensile strength and flexibility. Fourati et al. (2018) [28] observed similar phenomena in TPS/PBAT blends containing maleic anhydride and citric acid at levels up to 6%. These blends exhibited decreased TS (around 8 MPa) and EB (between 12 and 30%) due to significant disintegration between the polymer structure. This was characterized by the presence of voids and the agglomeration of TPS and PBAT particles in the cross-section micrograph of the film.

However, increased AP and SAP content increased YM values and were higher than pure TPS/PBAT blend (Figure 3c). This suggests that TPS played a significant role in the film properties. Wang et al. (2023) [8] demonstrated that increased PPC (amorphous alipathic polycarbonate) in the TPS phase of TPS/PBAT blend films exhibited better dispersion and compatibility, leading to an increased Young’s modulus.

### 3.4. Seal Strength

For practical applications, seals must be strong enough to withstand the impacts of normal handling and storage conditions. TPS/PBAT blend films can be heat-sealed at temperatures between 90 and 115 °C, with a holding pressure of 300 kPa and a dwell time of 1.5 to 2.0 s [21,22]. The sealing bands of the TPS/PBAT blend film containing AP and SAP exhibited smooth and wrinkle-free sealing bands at lower temperatures (95–105 °C). However, the seal area was weak and easily delaminated under manual pulling. The total quality of the sealing bands was not significantly affected by dwell times of 1.5 and 2.0 s. Based on the results of the experiments, the ideal sealing conditions for all film samples were chosen to be 110 °C with a holding pressure of 300 kPa and a dwell time of 1.5 s for subsequent sealing strength comparisons.

**Figure 4:**
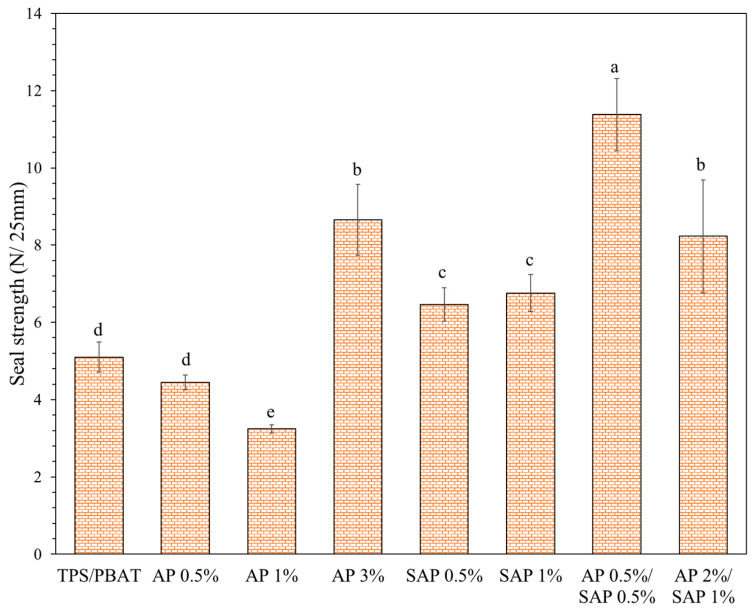
Seal strength properties of TPS/PBAT blend containing ascorbyl palmitate (AP) and sodium ascorbyl phosphate (SAP). Different lowercase letters (a–e) indicate significant difference (*p* ≤ 0.05) between samples.

The addition of AP reduced the seal strength of TPS/PBAT films, with a significant decrease observed at 1% AP concentration, as reported in Figure 4. In contrast, the incorporation of SAP increased the seal strength. AP showed a reduction in interfacial adhesion between polymers, leading to a decreased contact area of the heat seal and seal strength value. The ability of polymer adhesion influenced the increased contact area of the heat seal [41]. However, different behavior was observed for 3% AP and the 2% AP/1% SAP blend, where higher seal strength values were recorded. These films showed an agglomeration of granules from starch and high molecular AP interaction. The fraction of crystals or aggregates containing higher-molecular-weight components would melt and diffuse across the interface. These diffused chains formed more entanglements, contributing to greater seal strength [21,22,42].

### 3.5. Surface Wettability

**Figure 5:**
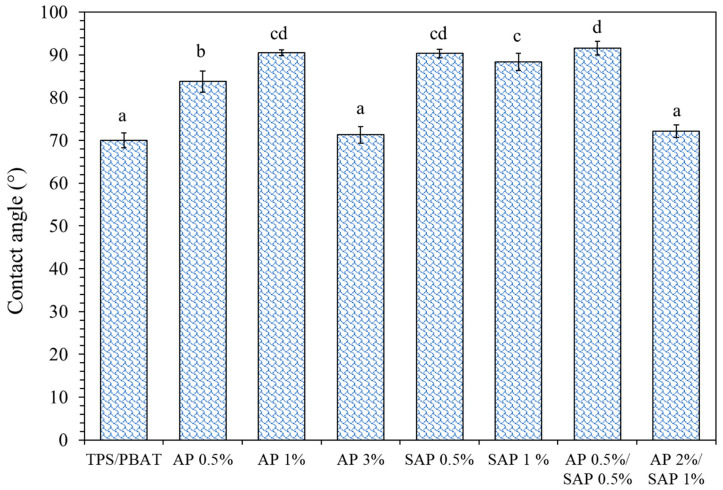
Contact angle of TPS/PBAT containing ascorbyl palmitate (AP) and sodium ascorbyl phosphate (SAP). Different lowercase letters (a–d) indicate significant difference (*p* ≤ 0.05) between samples.

The film surface’s hydrophilicity and hydrophobicity were evaluated using contact angle (CA) measurements, as shown in Figure 5. The pristine TPS/PBAT blend film exhibited an initial CA of 70.01°, reflecting the hydrophilic nature of TPS. Wang et al. (2023) [8] reported a decrease in CA from 90° to 78.9° for a TPS/PBAT film. The incorporation of AP and SAP resulted in an increased CA value. Notably, TPS/PBAT films containing AP 1%, SAP 0.5%, and the AP 0.5%/SAP 0.5% blend displayed CA values exceeding 90° (90.48°, 90.29°, and 91.55°, respectively). The presence of carbonyl groups by ascorbic acid of ascorbyl palmitate and sodium ascorbyl phosphate interacting with TPS via hydrogen bonding (as suggested by FT-IR) reduced the free hydroxyl group of TPS for bonding with water molecules. However, the highest concentration (3%) of AP and the blend (2% AP/1% SAP) exhibited insignificant differences in the CA value compared to the control film. This can be attributed to inhomogeneity within the matrix and the presence of pores. These factors likely resulted in a non-uniform distribution of wettability across the film surface, leading to a lower contact angle value.

### 3.6. Barrier Properties

**Figure 6:**
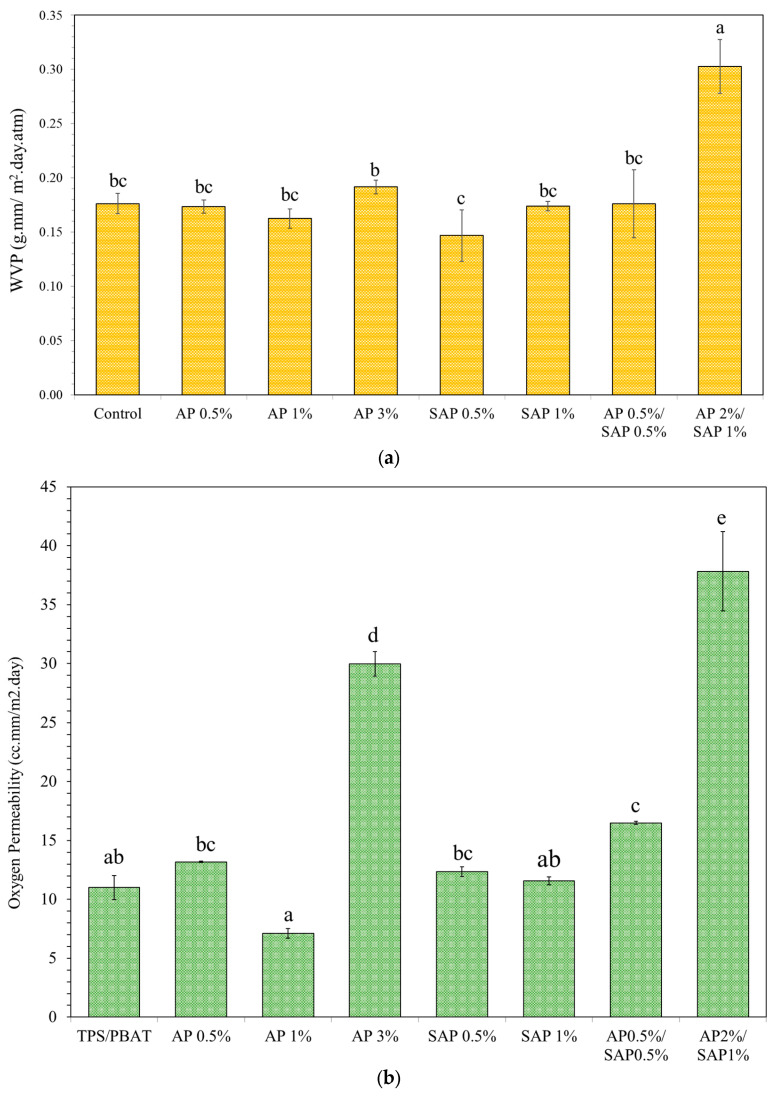
Barrier properties of (**a**) water vapor permeability and (**b**) oxygen of TPS/PBAT blend film containing ascorbyl palmitate (AP) and sodium ascorbyl phosphate (SAP). Different lowercase letters indicate significant difference (*p* ≤ 0.05) between samples.

The water vapor permeability (WVP) of the films showed insignificant changes with the addition of AP and SAP content (Figure 6a). The microstructures of the control films exhibited a PBAT-rich and TPS-rich phase which were smoother and rougher, respectively. The water vapor possibly transferred to the TPS phase was higher than PBAT due to more hydrophilicity and the presence of void spaces. The addition of AP and SAP produced numerous fine pores and the expansion of polar starch networks, which possibly enhanced the diffusion of water vapor [36]. However, the WVP insignificantly changed. It is presumed that a more homogeneous dispersion of TPS layers in the PBAT matrix possibly rearranged PBAT molecules, limiting water vapor transfer. Moreover, the WVP of the PBAT/TPS blends at a 50/50 ratio was possibly dominated by the PBAT matrix rather than the TPS phase. Accordingly, the modifying TPS phase by AP and SAP had no impact on the WVP values. Nevertheless, AP2%/SAP1% gave much higher WVP due to non-homogeneity and the pore structure in the films.

Similarly, the OP of the films with AP and SAP at 0.5% and 1% showed insignificant changes (Figure 6b). Oxygen, a diatomic non-polar molecule, diffuses through the polymer matrix influenced by the polar nature of the TPS phase [43]. Improved hydrophilicity of the matrix and increasing polarity were expected to reduce OP. However, the OP was possibly dominated by the PBAT phase. The AP3% and AP/SAP blends significantly increased OP, which was in concurrence with a great modification of the microstructures. AP0.5%/SAP0.5% showed particulate-like structures. Less connected polymeric matrices gave a higher level of micro-voids, which enhanced the permeation of volatile molecules [44].

### 3.7. Antioxidant Activity and UV Light Transmission

**Figure 7:**
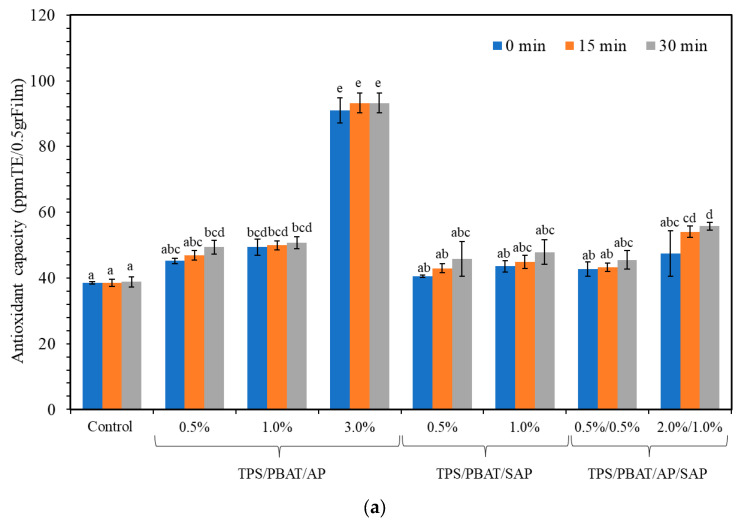

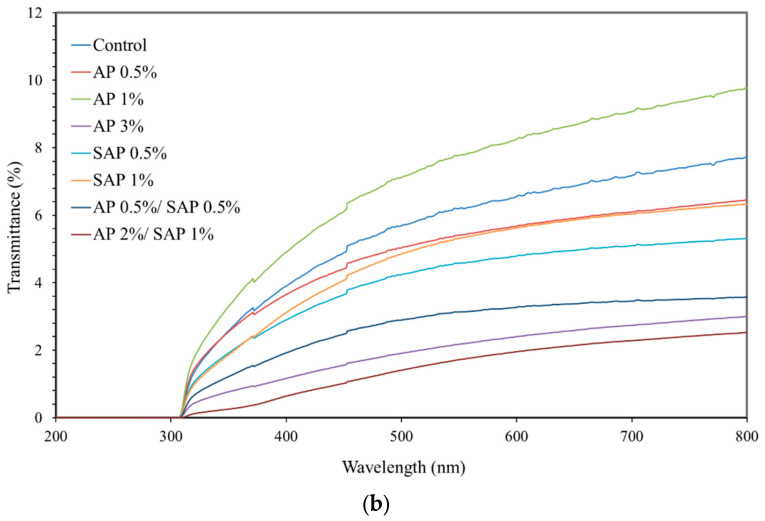
(**a**) Antioxidant activity; (**b**) UV light transmittance of TPS/PBAT film containing AP and SAP. Different lowercase letters indicate significant difference (*p* ≤ 0.05) between samples.

The antioxidant activity of the film samples, as measured by the DPPH assay in 95% ethanol, is presented in Figure 7a. Adding both AP and SAP enhanced the antioxidant capacity of the films. Carlotti et al. (2009) [45] studied the superior antioxidant efficacy of ascorbyl palmitate and sodium ascorbyl phosphate compared to phenylalanine against linoleic acid peroxidation. Increasing AP improved the antioxidant capacity. A sharply increasing antioxidant capacity was found in AP3%, suggesting a great release of antioxidant from the matrices. SEM indicated several large pores and void space in the matrices, which facilitated the diffusion and penetration of solvent. Accordingly, the AP greatly leached out, giving the highest antioxidant capacity. Films with SAP had a lower antioxidant capacity than AP (both 0.5% and 1%). A stronger interaction between SAP and the matrices possibly limited the release (as compared with AP), giving a lower antioxidant capacity.

UV radiation is a significant factor affecting food quality. The average percentage of UV light transmittance (%T) for the investigated TPS/PBAT blend films in the UV-C (200–280 nm), UV-B (280–315 nm), and UV-A (315–400 nm) regions is presented in Figure 7b. The results concluded that adding AP and SAP to TPS/PBAT blend films significantly reduced the film’s UV light transmittance values, enhancing their ability to block UV light. The best results were obtained by all samples, with the transmission percentage falling below 5% in the 200–400 nm wavelength range [46]. Nonetheless, the TPS/PBAT film’s light transmission increased with the addition of AP 1%. The smooth microstructure of AP 1% films was seen by SEM, indicating that the film’s uniform structure led to interaction between starch chains. AP gave a higher free volume by its ascorbic acid linkages between TPS and PBAT, leading to increased light transmission. Generally, TPS granules produce a window that lets UV light through, but PBAT functions as a semi-transparent barrier, absorbing part of the UV radiation and giving the area a white tint [7,14,34,46].

## 4. Conclusions

The TPS/PBAT blend films with AP and SAP were successfully produced by a blown-film extrusion process and demonstrated film compatibility. The addition of AP and SAP enhanced the adhesion between the TPS and PBAT polymer networks, resulting in a fiber-like microstructure and improved TPS dispersion. This study highlights the ability of AP and SAP to enhance the antioxidant capacity and UV-blocking properties of the films while maintaining their mechanical properties and barrier properties at appropriate concentrations. In particular, 1% AP and 0.5% SAP showed significant improvements in antioxidant activity and tensile properties, respectively, while the combination of 0.5% AP and SAP each provided balanced performance in all tests. However, at higher concentrations, non-homogeneous structures and voids between polymer blends led to increased permeability and decreased tensile strength, highlighting the trade-off between antioxidant uptake and structural integrity. These results indicate that AP and SAP can effectively enhance the antioxidant capacity and reduce UV transmittance, making the films suitable for biodegradable and antioxidant-enriched packaging solutions. Furthermore, these films have potential for preserving foods prone to oxidative degradation. Future research should investigate the long-term stability of these films under real-life conditions, the scaling up for industrial production, and the incorporation of other antioxidant systems to further optimize the film performance.

## Data Availability

The original contributions presented in the study are included in the article, further inquiries can be directed to the corresponding author.
